# Longitudinal characterization of diet-induced genetic murine models of non-alcoholic steatohepatitis with metabolic, histological, and transcriptomic hallmarks of human patients

**DOI:** 10.1242/bio.041251

**Published:** 2019-04-25

**Authors:** Naomichi Abe, Sayuka Kato, Takuma Tsuchida, Kanami Sugimoto, Ryuta Saito, Lars Verschuren, Robert Kleemann, Kozo Oka

**Affiliations:** 1 Sohyaku. Innovative Research Division, Mitsubishi Tanabe Pharma Corporation, 2-2-50, Kawagishi, Toda-shi, Saitama 335-8505, Japan; 2 Department of Microbiology and Systems Biology, Netherlands Organization for Applied Scientific Research (TNO), Utrechtseweg 48, 3700 AJ, Zeist, The Netherlands; 3 Department of Metabolic Health Research, Netherlands Organization for Applied Scientific Research (TNO), Zernikedreef 9, 2301 CE, Leiden, the Netherlands; 4 Department of Vascular Surgery, Netherlands Organization for Applied Scientific Research (TNO), Albinusdreef 2, P.O.Box 9600, 2333 ZA Leiden, The Netherlands

**Keywords:** Fast food, Fibrosis, Non-alcoholic steatohepatitis, Hyperinsulinemia, Obesity

## Abstract

Non-alcoholic steatohepatitis (NASH) is a fast-growing liver disease in the Western world. Currently, only a few animal models show both the metabolic and histological features of human NASH. We aimed to explore murine NASH models in a time dependent manner that exhibit metabolic, histological and transcriptomic hallmarks of human NASH. For this, the murine strains C57BL/6J, ob/ob, and KK-A^y^ were used and three types of nutritional regimes were administered: normal chow diet (NCD); high-fat, high-fructose, and high-cholesterol diet (fast food diet; FFD); or choline-deficient, L-amino acid-defined, high-fat diet (CDAHFD), for 2, 4, 8, 12, 18, 24, and 30 weeks. All strains under the FFD and CDAHFD regimes developed steatohepatitis. Among the strains treated with FFD, the non-alcoholic fatty liver disease (NAFLD) activity score, fibrosis progression and metabolic abnormalities such as hyperinsulinemia and obesity were more pronounced in ob/ob mice than in C57BL/6J and KK-A^y^ mice. In ob/ob mice fed FFD, the development of hepatic crown-like structures was confirmed. Furthermore, molecular pathways involved in steatohepatitis and fibrosis showed significant changes from as early as 2 weeks of starting the FFD regime. Ob/ob mice fed FFD showed metabolic, histological, and transcriptomic dysfunctions similar to human NASH, suggesting their potential as an experimental model to discover novel drugs for NASH.

## Abstract (precis)

**Summary:** We revealed that ob/ob mice under fast food diet (FFD) regime develop metabolic, histological and transcriptomic dysfunctions similar to human non-alcoholic steatohepatitis (NASH).

## INTRODUCTION

The number of patients with non-alcoholic steatohepatitis (NASH) has dramatically increased with the increase in the prevalence of obesity, type-2 diabetes and metabolic syndrome (Angulo, 2002; Sanyal, 2011; Saponaro et al., 2015). NASH is a growing cause of hepatocellular carcinoma (HCC) and liver transplantation, and its therapeutic management is increasingly important for preventing liver-related mortality (Ascha et al., 2010; Sanyal et al., 2006; Yatsuji et al., 2009). Recently ‘the multiple parallel hits hypothesis’ has been proposed as a mechanism underlying NASH progression. It is now clear that several factors such as insulin resistance, oxidative stress, mitochondrial dysfunction and inflammation contribute to the progression of steatohepatitis and fibrosis (Tilg and Moschen, 2010).

Obesity and type-2 diabetes have been clearly associated with NASH disease progression. Approximately 44% and 82% human NASH patients suffer from type-2 diabetes and obesity, respectively (Younossi et al., 2016). Obesity is positively correlated with both NAFLD prevalence and severity, and there is also a positive correlation between body mass index (BMI) and increased risk of NAFLD development (Loomis et al., 2016). High abdominal fat levels were also associated with the presence of biopsy-proven steatohepatitis (Margariti et al., 2015). In addition, insulin resistance is more prevalent in patients with NASH than in those with simple steatosis, suggesting that insulin resistance could accelerate NASH progression (Sanyal et al., 2001). Insulin resistance and hyperinsulinemia also may contribute to cell growth and progression to fibrosis (Inoue et al., 1999; Paradis et al., 2001). In patients with NAFLD, diabetes is an independent predictor of moderate-to-severe fibrosis (Hossain et al., 2009). These findings highlight the importance of obesity, hyperinsulinemia, and diabetes in NASH progression.

There are currently no approved drugs for NASH treatment (Filozof et al., 2015). One obstacle for drug development is the difficulty to develop preclinical models that show both the metabolic and histological features of human NASH (Santhekadur et al., 2018). For instance, the methionine and choline-deficient (MCD) diet-induced model has been frequently used in NASH research because it impairs the production of very-low density lipoproteins, resulting in steatosis, inflammation and advanced hepatic fibrosis. Thus, the MCD model could be useful for screening drugs that directly target hepatic fibrosis (Kajikawa et al., 2011). However, MCD diet decreases insulin levels and causes severe weight loss, indicating that metabolic characteristics of the MCD diet model are different from those in human NASH patients (Pickens et al., 2009). The severe weight loss typically observed in the MCD model suggests that it may not be suitable for evaluating medicines that target metabolic pathways. It has recently been reported that feeding mice with a choline-deficient, L-amino acid-defined, high-fat diet (CDAHFD), which is a modified choline-deficient diet containing 60 kcal % fat and 0.1% methionine, rapidly induces hepatic fibrosis and prevents body-weight loss in mice. This suggests that a CDAHFD-induced model might be more similar to human NASH than the MCD diet model. However, the CDAHFD model does not develop obesity (Matsumoto et al., 2013), although approximately 82% NASH patients in the US are obese (Younossi et al., 2016). Charlton et al. also reported administration of C57BL/6 mice with a high-fat, high-fructose and high-cholesterol diet (fast food diet; FFD) induces a NASH phenotype that not only includes steatohepatitis but also hyperinsulinemia, high glucose, and obesity (Charlton et al., 2011). However, this FFD model did not fully progress to severe steatohepatitis and advanced fibrosis, even after long-term (24 weeks) administration of FFD.

Taking these epidemiological and preclinical results into account, our hypothesis that a murine strain that develops obesity, hyperinsulinemia and diabetes, showing both metabolic and histological features of human NASH, could be useful for establishing NASH models. It is well known that ob/ob mice show hyperphagia due to leptin-deficiency, and as a result develop obesity and hyperinsulinemia. KK-A^y^ mice are generated by transferring the yellow obese gene (A^y^ allele) into KK/Ta mice. KK-A^y^ mice exhibit hyperphagia, obesity and hyperinsulinemia as well (Kennedy et al., 2010); however, these diabetic models administered a normal chow diet (NCD) regime do not fully progress to steatohepatitis and hepatic fibrosis (Larter and Yeh, 2008; Takahashi et al., 2012). On the other hand, ob/ob mice administered a high trans-fat, high-fructose, and high-cholesterol diet can develop hepatic fibrosis (Kristiansen et al., 2016; Trevaskis et al., 2012), suggesting that obesity, hyperinsulinemia and diabetes can exacerbate diet-induced steatohepatitis and fibrosis progression. Despite these findings, little has been reported on the sequence of events over time in diet-induced NASH models, and also a systematic comparison between diabetes-prone murine strains treated head-to-head with several NASH diets is lacking.

The purpose of this study was to explore and compare several murine NASH models exhibiting metabolic, histological and transcriptomic hallmarks of human NASH. We treated three murine strains (C57BL/6J, ob/ob and KK-A^y^ mice) with the NCD, CDAHFD and FFD regimes for up to 30 weeks, and characterized the events over time in these models by measuring metabolic and histological parameters. According to the observed translational character in these models we analyzed ob/ob mice treated with the FFD in more detail, and further characterized this model by assessing the development of hepatic crown-like structures (hCLS) and performing transcriptomic pathway analysis.

## RESULTS

### All strains administered FFD developed obesity compared with those fed NCD and CDAHFD

**Fig. 1.**
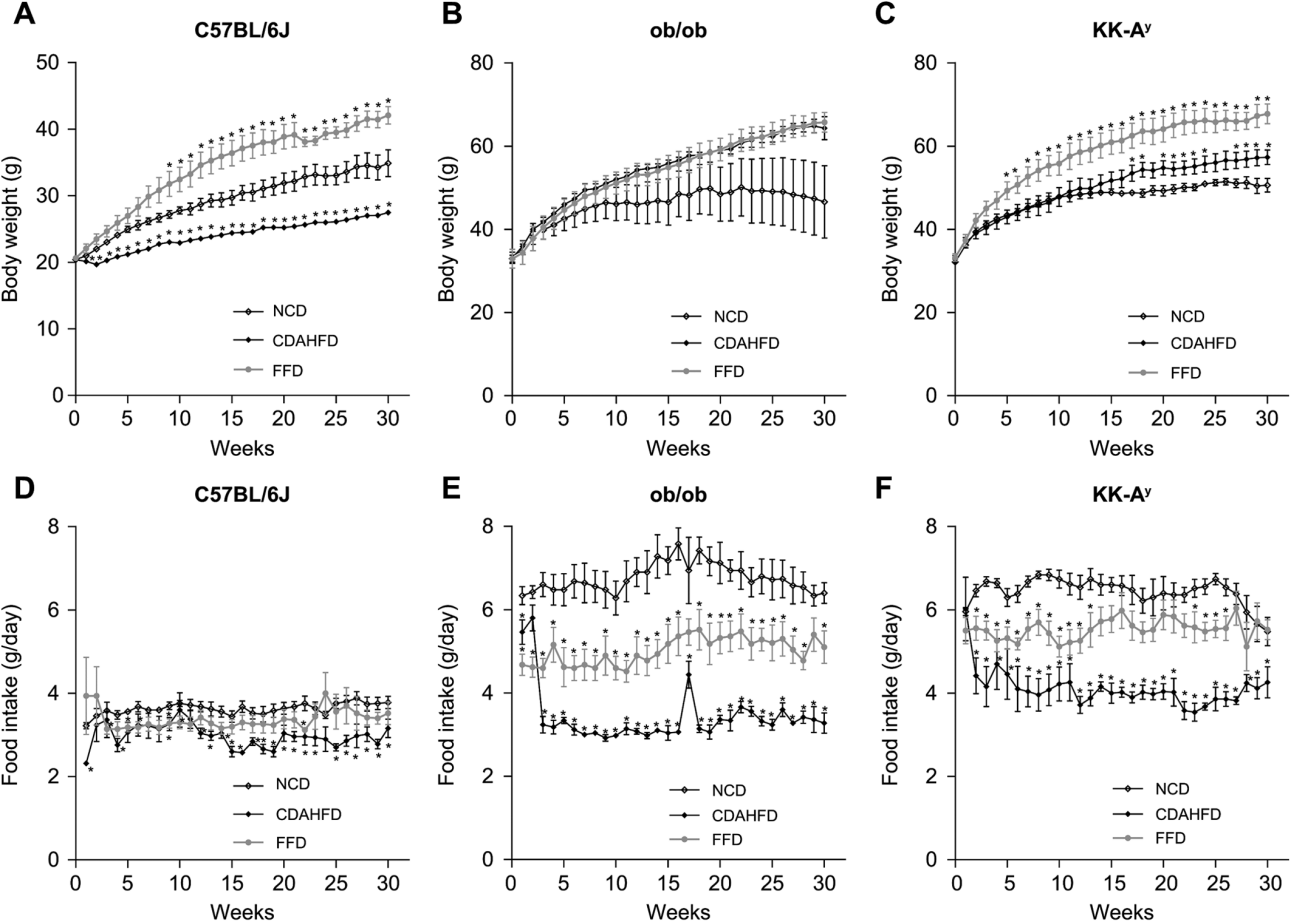
**Body weight and food intake changes in C57BL/6J, ob/ob and KK-A^y^ mice under NCD, FFD, or CDAHFD regimes for 30 weeks.** Body weight (A–C) and food intake (D–F). Values are mean±s.e.m., *n*=5. *P<0.05, **P<0.01 versus NCD (Student's *t*-test, two-tailed).

The C57BL/6J, ob/ob and KK-A^y^ mice fed FFD all gained more body weight than mice on the NCD regime, and developed obesity (Fig. 1A–C). When fed CDAHFD, ob/ob and KK-A^y^ mice developed obesity. However, the body weights were lower in C57BL/6J mice administered CDAHFD than in those fed NCD. Food intake of both ob/ob and KK-A^y^ mice was higher than in C57BL/6J mice (Fig. 1D–F). The FFD and CDAHFD regimes decreased food intake in ob/ob and KK-A^y^ mice compared to the NCD regime (Fig. 1D–F).

### FFD-fed ob/ob mice showed metabolic hallmarks of human NASH

**Fig. 2.**
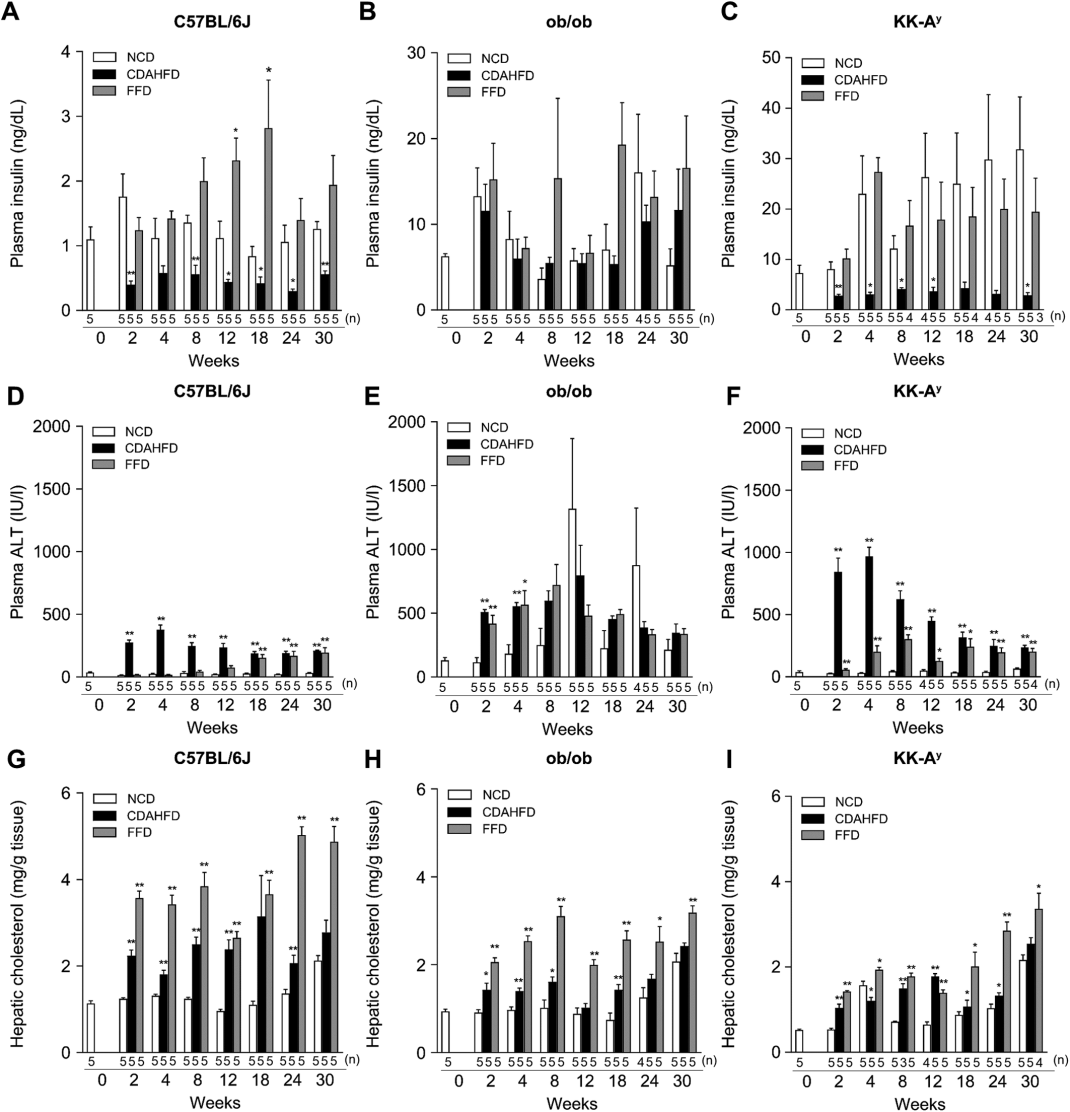
**Plasma insulin, plasma ALT levels and hepatic cholesterol levels in C57BL/6J, ob/ob and KK-A^y^ mice under NCD, FFD, or CDAHFD regimes for 2, 4, 8, 12, 18, 24 and 30 weeks.** Plasma insulin levels (A–C), plasma ALT levels (D–F) and hepatic cholesterol levels (G–I). Values are mean±s.e.m., *n*=3–5 (the exact number of animals are shown in the figure). *P<0.05, **P<0.01 versus NCD (Student's *t*-test, two-tailed).

The FFD regime significantly increased plasma insulin levels in C57BL/6J mice compared with the NCD regime at 12 and 18 weeks (Fig. 2A). The FFD regime also maintained hyperinsulinemia in ob/ob and KK-A^y^ mice (Fig. 2B,C). In contrast, the CDAHFD regime significantly decreased plasma insulin levels in the C57BL/6J and KK-A^y^ mice compared with the NCD regime, but it maintained hyperinsulinemia in ob/ob mice (Fig. 2A–C).

Plasma ALT levels in ob/ob mice were higher than in C57BL/6J mice (Fig. 2D,E). Compared with the NCD regime, plasma ALT levels were significantly increased in C57BL/6J and KK-A^y^ mice administered the CDAHFD regimes for 30 weeks (Fig. 2D,F). In C57BL/6J and KK-A^y^ mice, plasma ALT levels in FFD-fed groups were significantly higher than in the respective NCD-fed groups at 30 weeks (Fig. 2D,F). The FFD and CDAHFD regimes significantly increased plasma ALT levels in ob/ob mice at 2 and 4 weeks (Fig. 2E).

The FFD and CDAHFD regimes significantly increased hepatic cholesterol levels in all strains compared to the NCD regime (Fig. 2G–I).

Plasma glucose levels were significantly decreased after treatment with CDAHFD in all strains (Fig. S1A–C). The FFD regime significantly increased plasma glucose levels in C57BL/6J mice at 4, 8, and 12 weeks (Fig. S1A). Plasma glucose levels significantly decreased after treatment FFD in ob/ob mice (Fig. S1B). In KK-A^y^ mice, plasma glucose levels in the FFD-fed group were significantly increased at 2 weeks compared with in the NCD-fed group. However, the FFD regime significantly decreased plasma glucose levels at 12 weeks compared to the NCD regime in KK-A^y^ mice (Fig. S1C). The CDAHFD regimes significantly increased plasma ferritin levels in all strains at 30 weeks (Fig. S1D–F). In C57BL/6J and ob/ob mice, the FFD regime did not alter plasma ferritin levels (Fig. S1D,E). In KK-A^y^ mice, the FFD regime caused significant increases in plasma ferritin levels compared with the NCD regime at 30 weeks (Fig. S1F).

In C57BL/6J and ob/ob mice, the CDAHFD regime did not alter plasma triglyceride levels (Fig. S2A,B). In KK-A^y^ mice, plasma triglyceride levels in the CDAHFD-fed group were significantly decreased compared with in NCD-fed group at 2, 4 and 30 weeks (Fig. S2C). The FFD regime significantly decreased plasma triglycerides levels in C57BL/6J mice at 8 and 30 weeks and in KK-A^y^ mice at 2 and 12 weeks compared with the NCD regime (Fig. S2A,C). In ob/ob mice, plasma triglyceride levels in the FFD-fed group remained unchanged compared with in NCD-fed group (Fig. S2B).

There is a tendency that the FFD regime increased plasma cholesterol levels in all strains compared with the NCD regime (Fig. S2D–F). The CDAHFD regime significantly decreased plasma triglycerides levels in C57BL/6J mice and in KK-A^y^ mice at 2 and 4 weeks compared with the NCD regime (Fig. S2D,F). In all strains, there is a tendency that the FFD and CDAHFD regimes caused increases in hepatic triglyceride levels compared with the NCD regime (Fig. S2G–I).

Together with body-weight change, these results indicate that administration of the FFD regime in ob/ob mice induced metabolic abnormalities mimicking human NASH.

### FFD-induced steatohepatitis and fibrosis are accelerated in ob/ob mice compared to C57BL/6J and KK-A^y^ mice

**Fig. 3.**
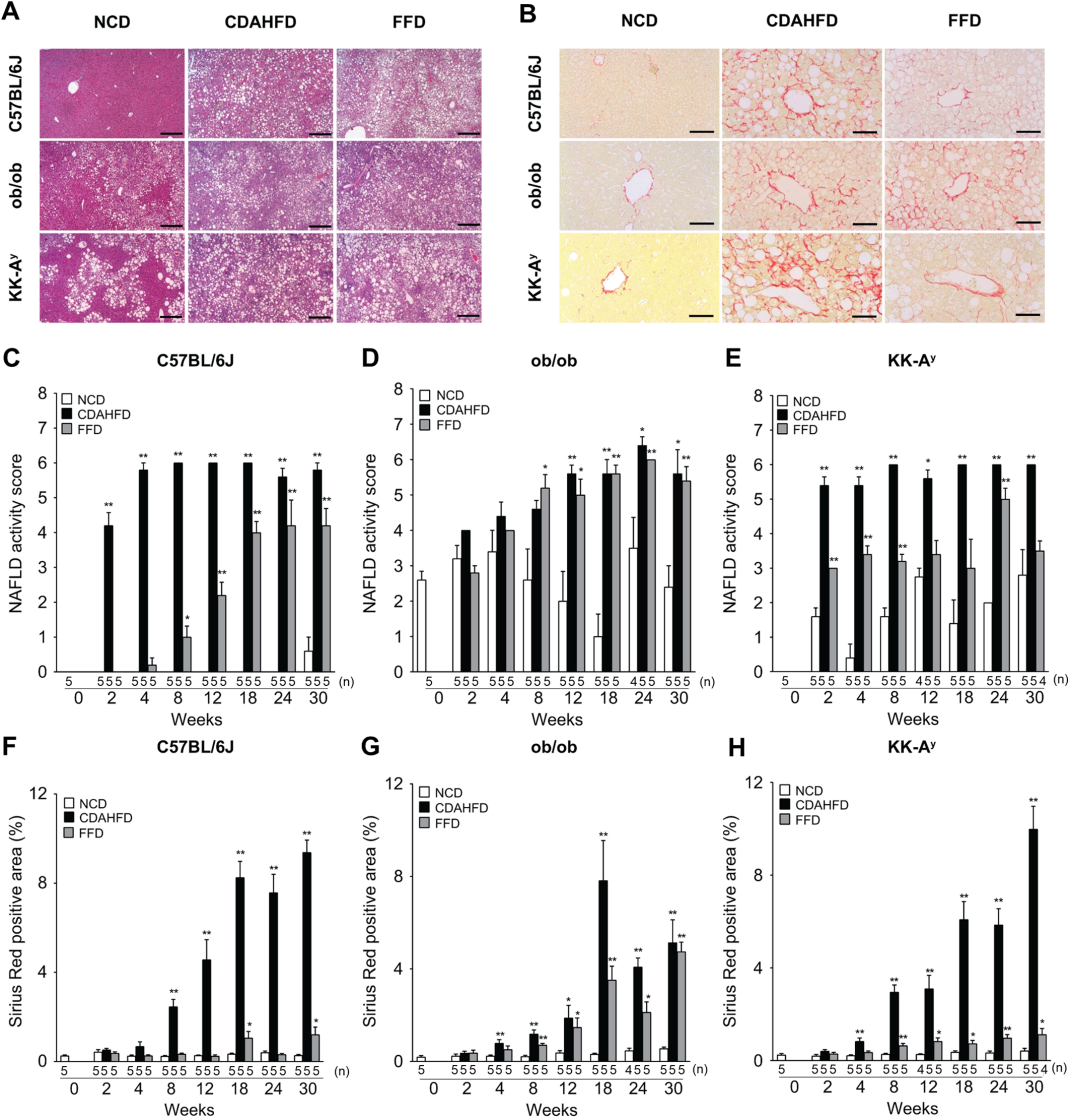
**Histological changes in C57BL/6J, ob/ob and KK-A^y^ mice under NCD, FFD, or CDAHFD regimes for 2, 4, 8, 12, 18, 24 and 30 weeks.** (A) Hematoxylin and Eosin staining of liver sections at 30 weeks. Scale bars: 200 µm. (B) Sirius Red staining of liver sections at 30 weeks. Scale bars: 50 µm. NAFLD activity score (NAS) (C–E) and Sirius Red-positive area (%) (F–H). Values are mean±s.e.m., *n*=4–5 (the exact number of animals are shown in the figure). *P<0.05, **P<0.01 versus NCD (NAS: Wilcoxon's test, Sirius Red-positive area: Student's *t*-test, two-tailed).

Representative pictures of HE and Sirius Red staining of all strains administered each of the regimes for 30 weeks are shown in Fig. 3A and B. Histological assessments of NAS and fibrosis as shown by the Sirius Red-positive area were performed at 2, 4, 8, 12, 18, 24 and 30 weeks. In all three strains, there is a tendency that both the FFD and the CDAHFD regimes increased the NAS compared with the NCD regime (Fig. 3C–E). The CDAHFD-fed groups developed a NAS of 5–6 points irrespective of the murine strain. The FFD regime induced a NAS of 3–4 points in C57BL/6J and KK-A^y^ mice, whereas it reached 5–6 points in ob/ob mice. In all three strains, both the FFD and the CDAHFD regimes significantly increased the Sirius Red-positive cross-sectional area compared with the NCD regime at 30 weeks (Fig. 3F–H). Among murine strains treated with FFD, fibrosis progression was more rapid and pronounced in ob/ob mice than in C57BL/6J and KK-A^y^ mice (Fig. 3F–H).

Components of the NAS are shown in Fig. S3. In all strains, the FFD and CDAHFD regimes significantly increased the steatosis score (Fig. S3A–C). Among murine strains treated with FFD, induction of inflammation was more rapid in ob/ob mice than in the C57BL/6J and KK-A^y^ mice (Fig. S3D–F). No ballooned hepatocytes were observed in all strains fed FFD or CDAHFD (Fig. S3G–I).

These results demonstrate that ob/ob mice under the FFD regime develop hallmarks of human NASH, such as obesity, hyperinsulinemia, elevated ALT levels, steatohepatitis and fibrosis. Therefore, we performed a further histological assessment and transcriptomic analysis in ob/ob mice fed FFD. We selected the FFD regime because the metabolic features of all strains administered this regime were more similar to human NASH than the CDAHFD regime. Furthermore, the histological changes, such as steatohepatitis and fibrosis, which were induced with the FFD regime, were more pronounced in ob/ob mice than in KK-A^y^ and C57BL/6J mice.

### FFD-fed ob/ob mice showed hepatic histological hallmarks of human NASH

**Fig. 4.**
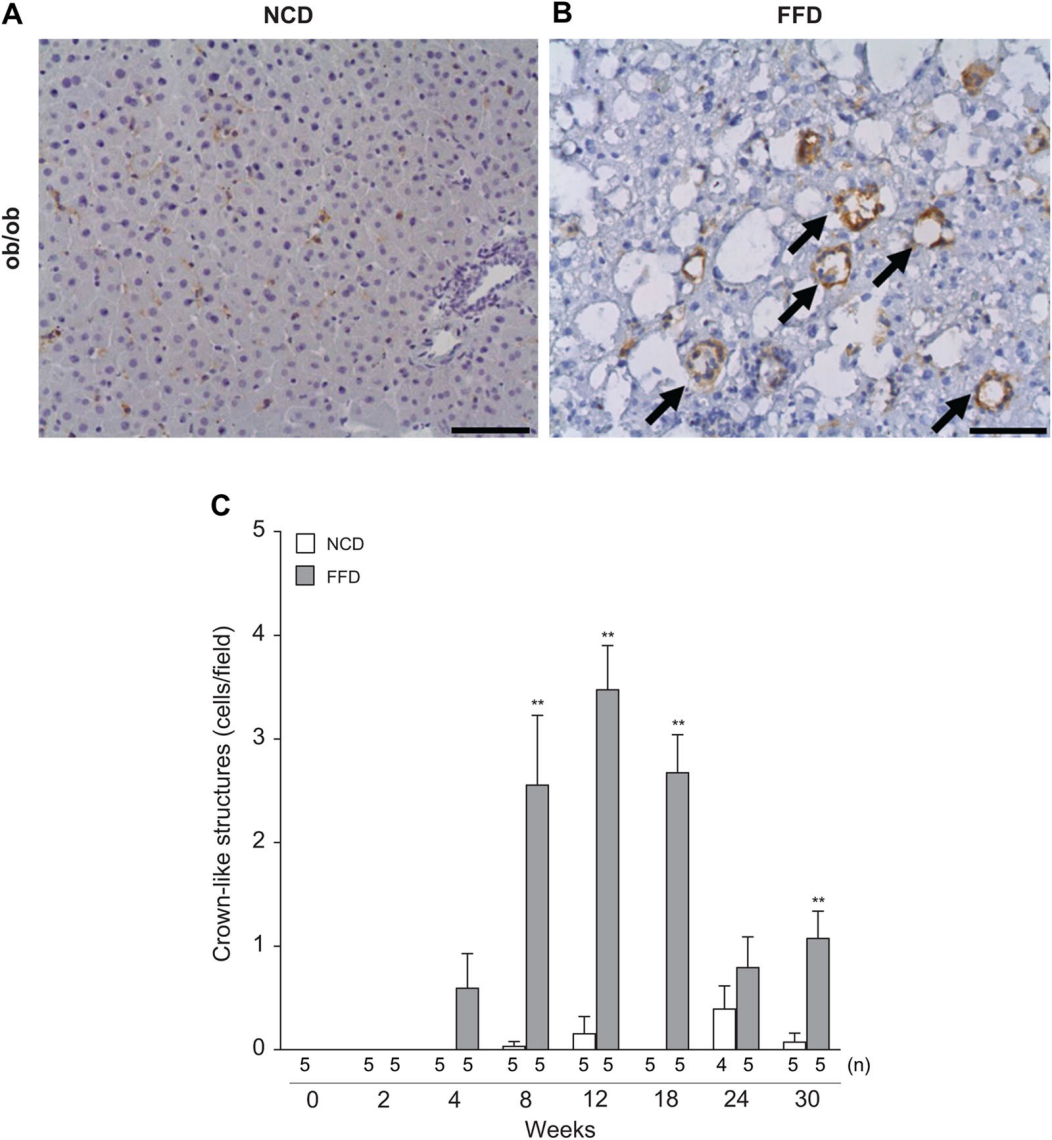
**Histological changes of hCLS in ob/ob mice under NCD or FFD regimes.** (A,B) F4/80 staining of liver sections at 12 weeks, (C) hCLS numbers (cells/field) at 2, 4, 8, 12, 18, 24 and 30 weeks. Values are mean±s.e.m., *n*=4–5 (the exact number of animals are shown in the figure). *P<0.05, **P<0.01 versus NCD (Student's *t*-test, two-tailed). Scale bars: 50 µm.

In the livers of NASH patients, macrophages are found in patterns termed ‘hepatic crown-like structures (hCLS)’ (Itoh et al., 2013). Representative pictures of F4/80 staining of FFD-fed ob/ob mice at 12 weeks are shown in Fig. 4A and B. The FFD regime significantly increased hCLS numbers in ob/ob mice compared with the NCD regime (Fig. 4C).

A general scoring system for rodent NASH models based on human NAS has recently been established (Liang et al., 2014). The components of the scoring system include microvesicular steatosis, macrovesicular steatosis, hypertrophic hepatocytes and inflammatory aggregates but not ballooned hepatocytes because these were only sporadically found in the livers of rodent NASH models. These four histological parameters have been commonly observed in both human patients and rodent NASH models (Liang et al., 2014; van Koppen et al., 2018). Ob/ob mice on the FFD regime developed a significant increase in macrovesicular steatosis, inflammation and hypertrophy compared with the NCD regime (Fig. S4A–D).

These results suggest that ob/ob mice fed FFD develop histological hallmarks of human NASH, although no ballooned hepatocytes were observed.

### Disease progression-related pathways were significantly changed in FFD-fed ob/ob mice

**Fig. 5.**
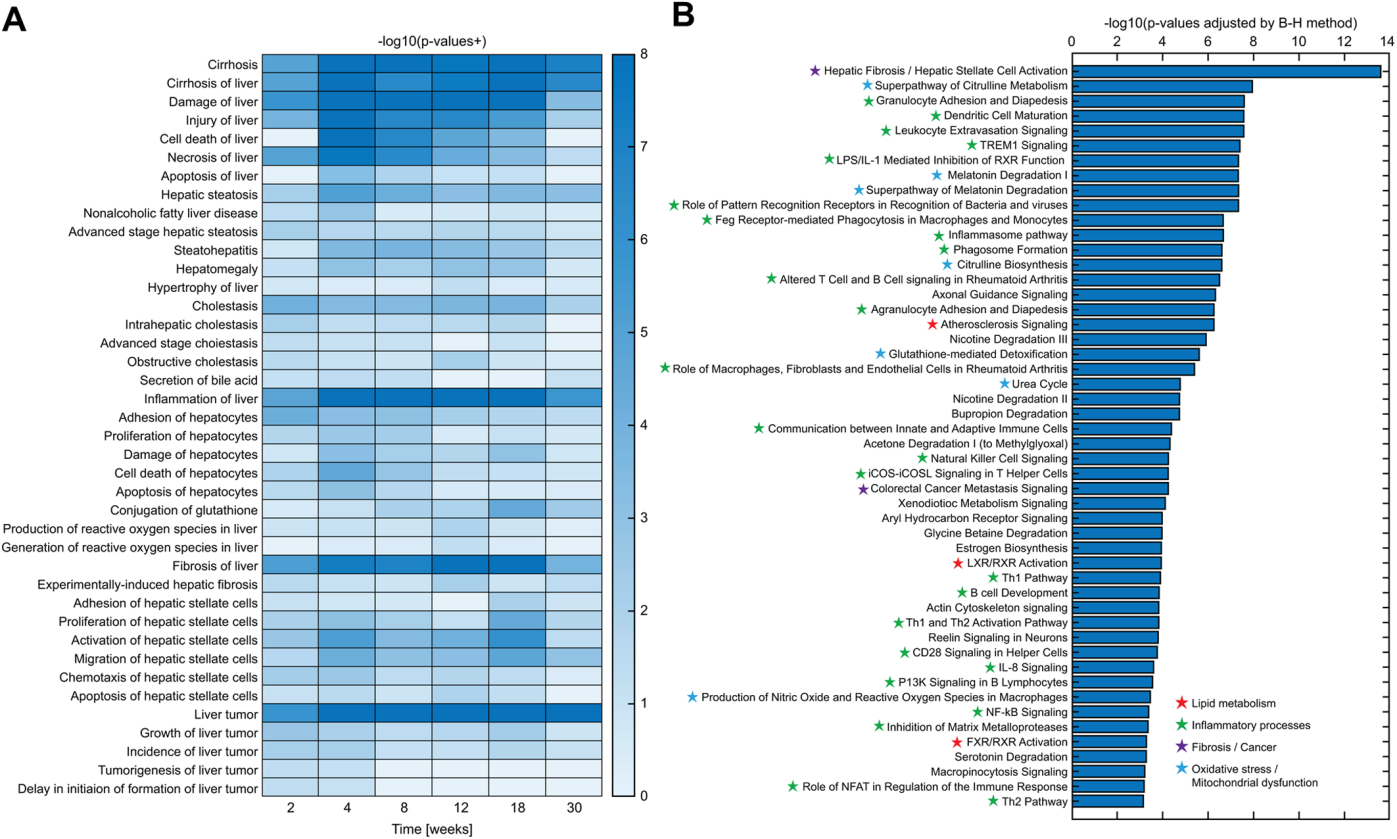
**Hepatic transcriptomic pathway analysis in ob/ob mice under NCD or FFD regimes.** (A) Heat map visualization of biological categories related to NASH disease progression for 30 weeks. Values are expressed as–log (*P*-values). (B) Top 50 canonical pathways at 18 weeks. Red stars, green stars, purple stars, and blue stars indicate pathways involved in lipid metabolism, inflammatory processes, fibrosis and cancer and oxidative stress, respectively.

To investigate the underlying mechanisms of disease development we performed a gene expression analysis in the livers of ob/ob mice fed FFD, using the method of next generation RNA sequencing. The heat map of the biological categories is shown in Fig. 5A. Inflammation, fibrosis, tumor and cirrhosis pathways were significantly activated in ob/ob mice on the FFD regime. Major pathways involved in disease progression were further analyzed using the IPA software; the top 50 canonical pathways at 18 weeks are listed in Fig. 5B. The processes of both hepatic fibrosis and stellate cell activation were strongly activated. Furthermore, pathways involved in inflammatory processes and oxidative stress were significantly affected by the FFD regime. Super-pathways of citrulline metabolism, citrulline biosynthesis and urea cycle pathways were also significantly downregulated, suggesting mitochondrial dysfunction in FFD-fed ob/ob mice.

### FFD-fed ob/ob mice develop rapid gene expression changes characteristic for inflammation and fibrosis in the liver

**Fig. 6.**
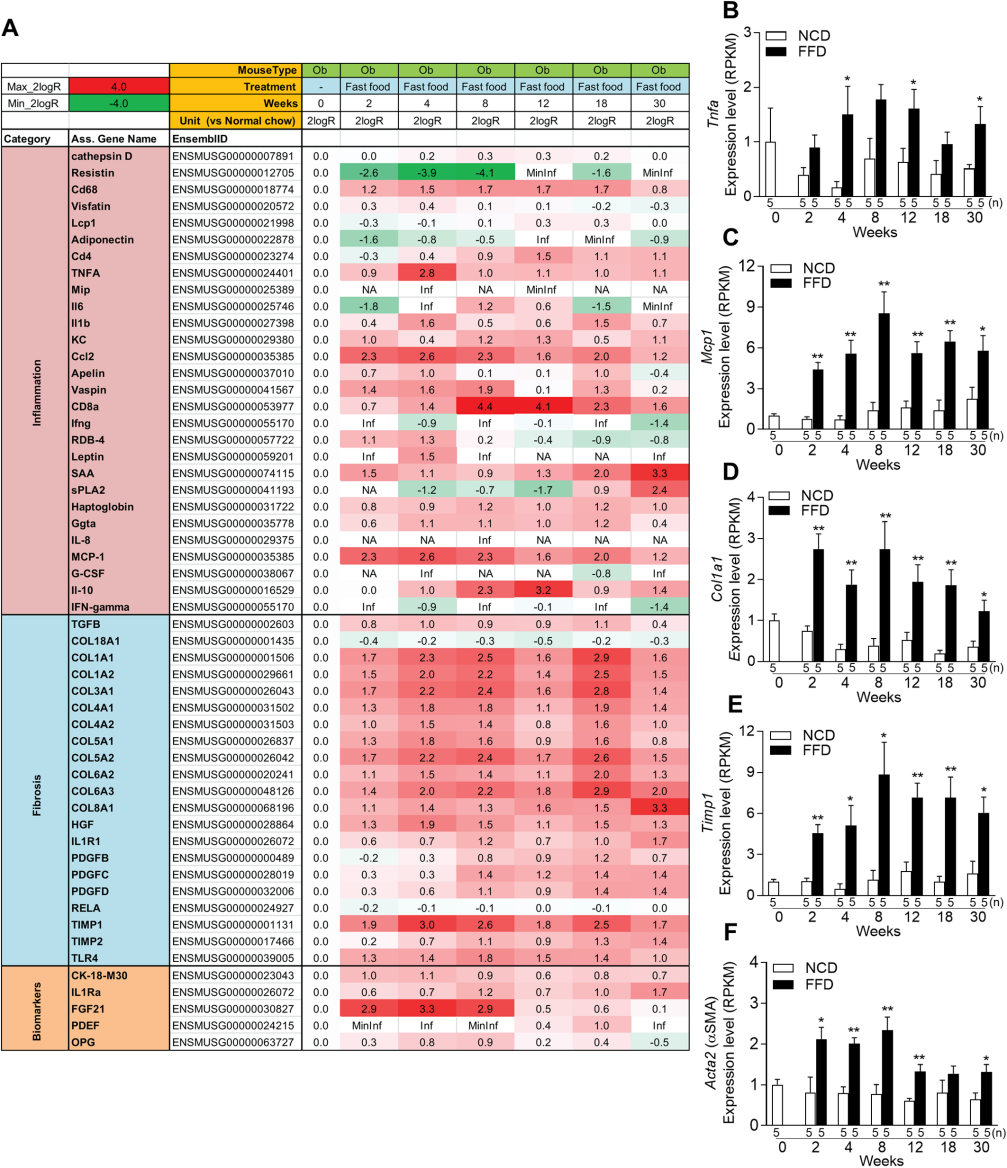
**Hepatic gene expression analysis in ob/ob mice under NCD or FFD regimes for 2, 4, 8, 12, 18, 24 and 30 weeks.** (A) Heat map of genes involved in inflammation and fibrosis. Red, upregulated; green, downregulated. (B–F) Expression levels of selected genes. Values are mean±s.e.m., *n*=5. *P<0.05, **P<0.01 versus NCD (Student's *t*-test, two-tailed).

Fig. 6A shows a heat map of genes that were upregulated or downregulated in the livers of ob/ob mice under the FFD regime compared with ob/ob mice fed NCD. The FFD regime resulted in a rapid induction of pro-inflammatory and pro-fibrotic genes after only 2 weeks. The expression levels of *Tnf*-α, *Mcp*-1, *Col1a1*, and *Timp*-1, *Acta2* (αSMA) as representative genes involved in inflammation and fibrosis, are also shown as bar graphs (Fig. 6B–F). Increased expression of these genes compared to their time-matched control groups was observed until the end of the study at 30 weeks indicating that the pro-inflammatory and pro-fibrotic condition persisted.

These results suggest that the FFD regime rapidly increased the expression of genes involved in inflammation and fibrosis in ob/ob mice compared with the NCD regime, and precedes histological manifestation of steatohepatitis and fibrosis.

## DISCUSSION

In this study, we treated three murine strains (C57BL/6J, ob/ob and KK-A^y^) with NCD, CDAHFD and FFD for 30 weeks, and we found that FFD-fed ob/ob mice exhibited metabolic and histological hallmarks of human NASH. All strains developed steatohepatitis and fibrosis after being administered CDAHFD and FFD. However, the metabolic features differed among strains administered these diet regimes. The FFD regime not only exacerbated histological features of NASH but also induced metabolic abnormalities such as obesity and hyperinsulinemia in all strains. Obesity and hyperinsulinemia were only observed in ob/ob mice under the CDAHFD regime. By contrast, the CDAHFD regime attenuated and improved the metabolic phenotypes in C57BL/6J and KK-A^y^ mice. Thus, the FFD regime could be more suitable for establishing NASH models with hallmarks of human NASH compared to the CDAHFD regime.

In CDAHFD-fed mice, metabolic and histological phenotypes were strain-dependent, suggesting that the genetic background of each strain is a key determinant of NASH disease progression. It is well known that a choline-deficient diet results in body-weight loss and decreased plasma insulin levels (Pickens et al., 2009; Rinella et al., 2008). The consequences of body-weight changes and plasma insulin levels in CDAHFD-fed C57BL/6J mice in this study were consistent with those in the previous report (Matsumoto et al., 2013). However, CDAHFD-fed ob/ob and KK-A^y^ mice gained body weight. Although plasma insulin levels were significantly decreased in CDAHFD-fed KK-A^y^ mice, this dietary regime did not affect hyperinsulinemia in ob/ob mice. Thus, CDAHFD-fed ob/ob mice replicated some hallmarks of human NASH and could be useful for preclinical drug testing as is the case for the FFD-fed ob/ob mice. However, it is unclear why ob/ob strain, which is a genetic one with obesity and hyperinsulinemia, is more resistant regarding the observed improvements of metabolic parameters upon CDAHFD administration, compared with C57BL/6J mice. Further studies are needed to reveal the underlying mechanisms of diverse phenotypes induced by the CDAHFD regime among strains.

Overall, FFD regime maintained or increased body weight and plasma insulin levels in all strains even though there is not a statistical significance, but a tendency at some time points, presumably due to relatively small number of mice per group per time point, which is a limitation of the study. Furthermore, FFD-fed ob/ob mice exhibited more rapid and severe progression of steatohepatitis and fibrosis than FFD-fed C57BL/6J and KK-A^y^ mice. Steatosis and inflammation scores of NAS in ob/ob mice were higher than in the C57BL/6J and KK-A^y^ mice at baseline of week 0, suggesting that genetic features associated with steatosis and inflammation in ob/ob mice could accelerate diet-induced steatohepatitis and fibrosis. It has been reported that ob/ob mice fed a high trans-fat diet developed severe hepatic fibrosis compared with C57BL/6J mice fed a high trans-fat diet (Kristiansen et al., 2016). Taken together these results suggest that ob/ob mice could be more susceptible to diet-induced steatohepatitis and fibrosis progression than other strains.

Rapid induction of pro-inflammatory and pro-fibrotic genes was observed after only 2 weeks of the FFD-fed in ob/ob mice, a time point at which histological changes had not yet become obvious. In terms of preclinical drug testing, the expression of genes involved in inflammation and fibrosis could be a useful early readout in the FFD-fed ob/ob model. In addition, the molecular pathways involved in oxidative stress, inflammation, stellate cells activation processes, mitochondrial dysfunction and HCC development were all significantly changed in the FFD-fed ob/ob mice between 2 and 30 weeks. In this study, we observed that the expression levels of αSMA gene, a marker of hepatic stellate cell activation, were significantly increased in the FFD-fed ob/ob mice compared with NCD-fed controls. Consistent with our results on gene expression level, Krishnan and coworkers showed by immunohistochemistry that the αSMA-positive area in livers of FFD-fed C57BL/6J mice was significantly increased compared with NCD-fed C57BL/6J mice (Krishnan et al., 2017).

The FFD regime contained high-fat, high-fructose, and high-cholesterol (i.e. 2% w/w). Free fatty acids are known as the trigger of mitochondrial dysfunction, oxidative stress and activation of hepatic stellate cells (Day, 2002). Fructose is known as a major driving factor of lipotoxicity in the liver, leading to NAFLD development and progression (Jensen et al., 2018). For instance, consuming fructose, rather than glucose, increased visceral adiposity and decreased insulin sensitivity in obese patients (Stanhope et al., 2009). Fructose consumption has been found to be higher in patients with NAFLD than in age-matched controls, and increased fructose consumption has been associated with fibrosis severity in patients with NAFLD (Abdelmalek et al., 2010). In addition, it has been reported that free cholesterol directly activates hepatic stellate cells, suggesting that the high cholesterol in the FFD regime promotes NASH disease progression as well (Tomita et al., 2014). Thus, we infer that the excess amount of fat, fructose and cholesterol in FFDs exacerbates a lipotoxic environment and accelerates progression of steatohepatitis and fibrosis in ob/ob mice.

In this study, hepatic fibrosis in leptin-deficient ob/ob mice was more pronounced than in the C57BL/6J and KK-A^y^ mice when administered the FFD regime. Leptin has been reported to be a key factor in the activation of hepatic stellate cells and fibrosis progression. Liver fibrosis in ob/ob mice was milder than in normal mice administered a CCl_4_ injection or with a MCD diet (Leclercq et al., 2002; Sahai et al., 2004). We surmise that obesity and hyperinsulinemia induced by leptin deficiency, rather than the activation of hepatic stellate cells by leptin, might make a greater contribution to steatohepatitis and subsequent fibrosis progression in ob/ob mice fed FFD.

Despite the wide use of an NAFLD activity scoring system for the evaluation of disease progression in patients and drug effects in clinical trials, it has been reported that ballooned hepatocytes were occasionally observed in several preclinical animal studies (Kleiner et al., 2005; Liang et al., 2014). However, no ballooned hepatocytes were observed in any mice groups in this study. Our results were consistent with previous reports showing that no ballooned hepatocytes were observed in FFD-fed C57BL/6J mice (Krishnan et al., 2017). These preclinical results suggest that NAS might be in particular be suitable for human samples and the human scoring systems needs to be adapted for use in experimental rodents as detailed in a study comparing human and murine histopathology (Liang et al., 2014; Morrison et al., 2018b). Although ballooned hepatocytes were not observed, FFD-fed ob/ob mice showed macrovesicular steatosis, microvesicular steatosis, hypertrophy and inflammatory aggregates in their livers, which have been reported as the common histological hallmarks of human and rodent NASH (Liang et al., 2014). In addition, the number of hCLS, which is composed of macrophages surrounding damaged hepatocytes, was significantly increased during disease progression in the FFD-fed ob/ob mice. hCLS was reported as a histological hallmark in patients with NASH and was significantly increased in a NASH model of melanocortin-4 receptor deficient mice fed a Western diet (Itoh et al., 2013). hCLS numbers were positively associated with the NAS of ballooning and was higher in NASH patients with stage-2 fibrosis compared with those having less than a stage-1 fibrosis (Itoh et al., 2013). Cholesterol crystals in hepatocytes and Kupffer cells have been associated with an increase in hCLS numbers (Ioannou et al., 2015). We speculate that administration of the FFD regime, which contains 2% w/w of cholesterol, could significantly increase hepatic cholesterol levels and result in the appearance of hCLS. Therefore, histological features observed in ob/ob mice fed the FFD regime mimic several of the histological characteristics of human NASH patients.

There are several limitations in this study. First, since the aim of this study was to longitudinally characterize the FFD-, and CDAHFD- induced NASH models in three strains and to follow up the histological features by euthanizing them at each time point for 30 weeks, the used mouse numbers were relatively small, five mice per group per each time point. Although it might not be a large enough number in terms of statistical analysis, we could assess the characteristics of each NASH model including the variations of biochemical and histological parameters. As a result, we identified FFD-treated ob/ob mice as a model that develops metabolic, histological and transcriptomic similarities when compared to human NASH. In order to assess the value FFD-treated ob/ob model for compound testing, it is important to validate the model with established compounds in future studies (e.g. tool compounds or drugs in clinical such as obeticholic acid), and to compare transcriptomics and metabolomics disease profiles with human disease profiles, essentially as we have shown for Ldlr−/−. Leiden mice, a translational NASH models developing a severely obese and hyperinsulinemic phenotype (Morrison et al., 2018a,b). A second limitation is that the gene expression and pathway analysis of CDAHFD-fed models was not performed in this study. Transcriptomic comparison of FFD- and CDAHFD-fed models could provide additional information for further characterization. However, we lowered the priority of the pathway analysis of CDAHFD-fed model because the metabolic phenotype (e.g. plasma insulin levels) is not similar to human patients, both in CDAHFD-fed C57BL/6J mice and CDAHFD-fed KK-A^y^ mice.

In conclusion, the FFD-fed ob/ob mice develops metabolic, histological and transcriptomic hallmarks of human NASH. Therefore, ob/ob mice fed FFD could be a useful preclinical model for drug testing which will require more refined molecular profiling and validation studies using compounds as described recently (Morrison et al., 2018b). In addition, our results suggest that a genetic deletion favoring obesity and hyperinsulinemia (leptin-deficiency) accelerates steatohepatitis and fibrosis progression.

## MATERIALS AND METHODS

### Animals

Male C57BL/6J and C57BL/6J Ham Slc-ob/ob mice (age 5 weeks) were obtained from Japan SLC, Inc. (Tokyo, Japan). In addition, male KK-A^y^/TaJcl mice (age 5 weeks) were purchased from CLEA Japan, Inc. (Tokyo, Japan). All mice were housed in a 12/12 h dark/light cycle environment. Room temperature was controlled to 22°C±3°C, with 50%±20% humidity. Experimental protocols concerning the use of laboratory animals were reviewed and endorsed by the Institutional Animal Care and Use Committee.

### Dietary interventions

At age 6 weeks, the three different strains of mice were administered three different nutritional regimes: [A] NCD (CRF1, oriental, Tokyo, Japan) or [B] a high-fat (41 kcal %), high-fructose (30 kcal %), and high-cholesterol (2% w/w) diet (FFD, D12042201, Research Diet, USA), (Charlton et al., 2011) or [C] a high-fat (62 kcal %), choline-deficient and 0.1% methionine diet (CDAHFD, A06071302, Research Diet, NJ, USA) (Matsumoto et al., 2013). These regimes were continued for 2, 4, 8, 12, 18, 24 and 30 weeks, and body weight and calorie intake were measured during this period. Three to five mice per regime at each time point were euthanized, and liver and plasma samples were collected for histological and biochemical analysis.

### Plasma biochemical analysis

Plasma levels of alanine aminotransferase (ALT), aspartate aminotransferase (AST) and glucose were measured using an auto analyzer HITACHI 7070 (HITACHI, Tokyo, Japan). Plasma levels of insulin were measured using the mouse insulin quantification enzyme-linked immunosorbent assay (ELISA) Kit (Morinaga, Tokyo, Japan). Plasma levels of ferritin were measured using mouse ferritin quantification ELISA Kit (GenWay Biotech Inc., CA, USA). Hepatic triglycerides and hepatic total cholesterol were measured using the Test Wako kit (Wako, Tokyo, Japan). All measurements were performed according to the manufacturer's instructions for each kit.

### Liver histological analysis

Liver samples were collected from the left lateral lobe. They were fixed in 10% formalin, paraffin-embedded, and sectioned (4-μm thickness) using a microtome (RM2255, Leica microsystems, Wetzlar, Germany). For evaluating NAFLD activity scores (NAS) (Brunt et al., 2011) and the Sirius Red-positive area, each liver section was stained with Hematoxylin and Eosin (HE) and Sirius Red, respectively. The Sirius Red-positive area was quantified using ImageJ software (National Institute of Health, MD, USA). Macrovesicular steatosis, microvesicular steatosis, hypertrophy and inflammatory cell aggregates in the livers of FFD-fed ob/ob mice at all time points were assessed according to a previously described scoring method (Liang et al., 2014). To assess the development of hCLS, our immunohistochemistry analyses utilized the anti-F4/80 antibody (T-2028, BMA Biomedicals, Augst, Switzerland) as a primary antibody and goat anti-rat immunoglobulin G (IgG) as the secondary antibody (62-9520, Thermo Fisher Scientific Inc., MA, USA).

### Hepatic ribonucleic acid (RNA) isolation, gene expression, and molecular pathway analysis by RNAseq

Total RNA was extracted from the livers of both ob/ob mice fed NCD and those receiving FFD at all time points, using the Ambion RNAqueous total RNA isolation kit (Thermo Fisher Scientific). RNA concentrations were determined spectrophotometrically using NanoDrop 1000 (Isogen Life Science, De Meern, the Netherlands), and RNA quality was assessed using the 2100 Bioanalyzer (Agilent Technologies, Amstelveen, the Netherlands). Strand-specific mRNA-seq libraries for the Illumina platform were generated and sequenced at GenomeScan (Leiden, the Netherlands). The libraries were multiplexed, clustered, and sequenced on an Illumina HiSeq 2500 using a single-read 75-cycle sequencing protocol, with 15 million reads per sample, and indexed. The RPKM calculation method we used has been previously described (Mortazavi et al., 2008). Differentially expressed genes (DEGs) between NCD regime and FFD groups were determined using the DEseq-method with the statistical cut-off at false-discovery rate (FDR) of <0.05. DEGs were used as an input for pathway analysis with the use of the Ingenuity Pathway Analysis (IPA) suite (www.ingenuity.com, accessed 2016). The method of statistical analysis and *P*-value calculations using DEseq has been previously described (Anders et al., 2013).

### Statistical analyses

All data were expressed as mean±s.e.m. The study primarily aimed at characterizing the longitudinal development of NAFLD in response to FFD and CDAHFD. Hence two statistical comparisons were considered being most relevant, FFD versus NCD and CDAHFD versus NCD. The identification of the most appropriate diet was taken on basis of an inducing or lowering effect on metabolic risk factors (e.g. increase or decrease of fasting insulin). Comparison of mean values between two groups (FFD versus NCD or CDAHFD versus NCD) was performed using Student's *t*-test. The comparisons of NASs were performed using Wilcoxon's test. *P*-values of <0.05 were considered statistically significant.

## Supplementary Material

Supplementary information
