# Supplementary material for: Longitudinal characterization of diet-induced genetic murine models of non-alcoholic steatohepatitis with metabolic, histological, and transcriptomic hallmarks of human patients
Source: Biol Open. 2019 Apr 25;8(5):bio041251. doi: 10.1242/bio.041251 (PMC6550083; doi:10.1242/bio.041251)
Supplement: Supplementary information [file biolopen-8-041251-s1.pdf]

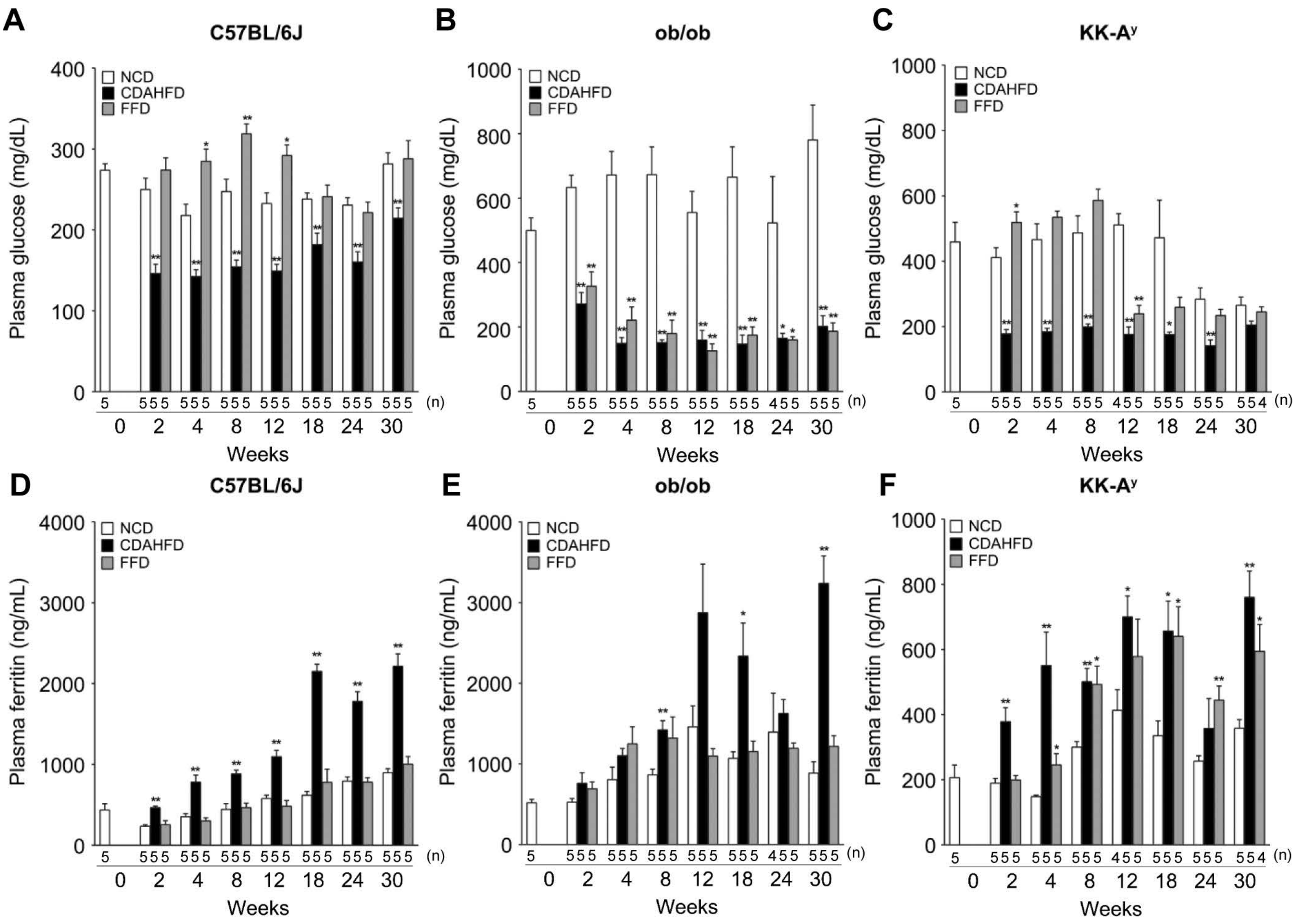

Fig. S1. Plasma glucose levels and plasma ferritin levels in C57BL/6J, ob/ob, and KK-A^y mice under NCD, FFD, or with a CDAHFD regimes for 2, 4, 8, 12, 18, 24, and 30 weeks. (A), (B), and (C) Plasma glucose levels (D), (E), and (F) Plasma ferritin levels. Values are mean ± SEM, n = 4–5 (the exact number of animals are shown in the figure). $^{*}p < 0.05$, $^{**}p < 0.01$ vs. NCD (Student's t-test, two-tailed).

Biology Open • Supplementary information

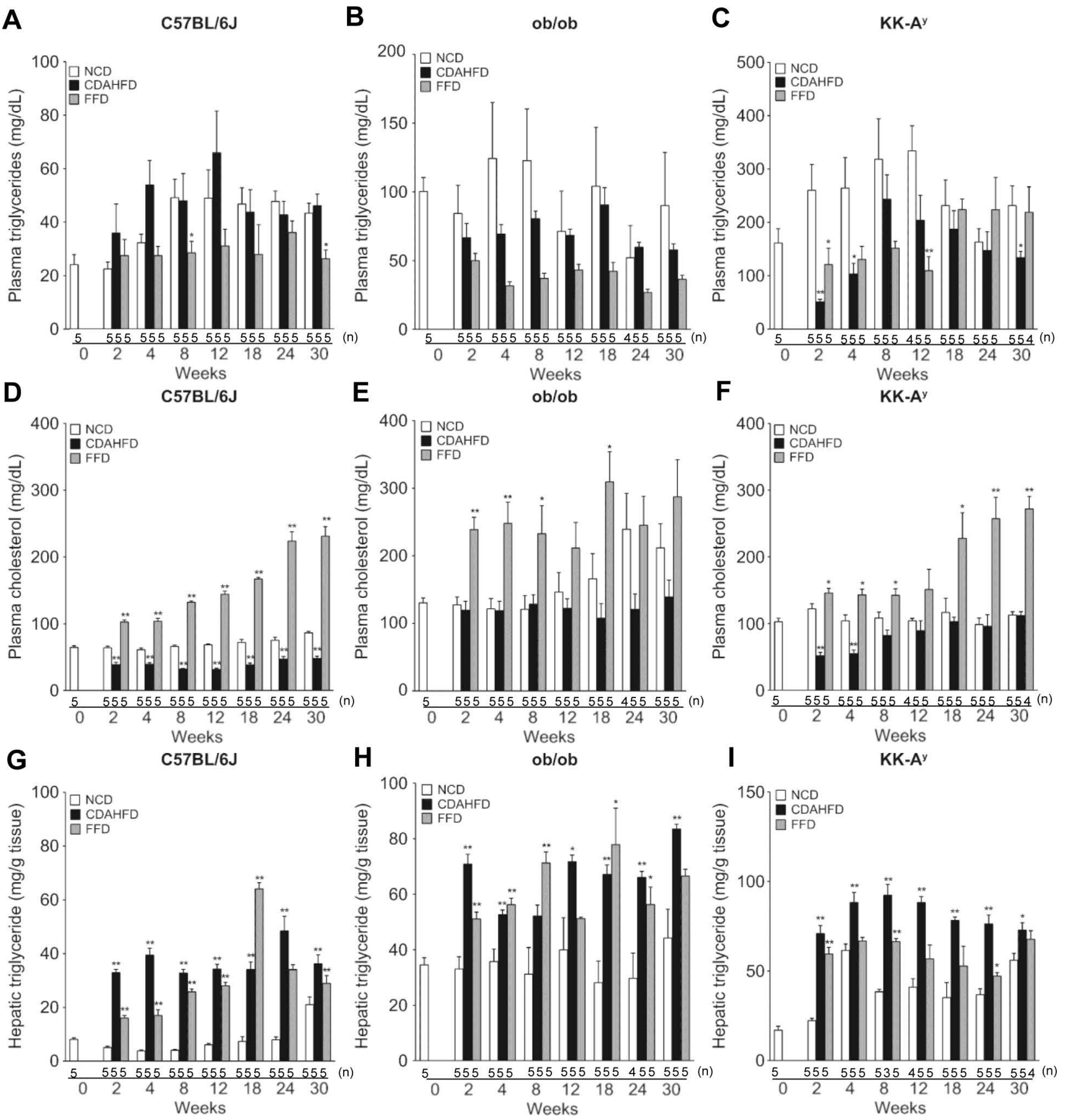

Fig. S2. Plasma triglyceride levels, plasma cholesterol levels, and hepatic triglyceride levels in C57BL/6J, ob/ob, and KK-A^{y} mice under NCD, FFD, or CDAHFD regimes for 2, 4, 8, 12, 18, 24, and 30 weeks. (A), (B), and (C) Plasma triglyceride levels (D), (E), and (F) Plasma cholesterol levels (G), (H), and (I) Hepatic triglyceride levels. Values are mean ± SEM, n = 3–5 (the exact number of animals are shown in the figure). $^{*}p < 0.05$, $^{**}p < 0.01$ vs. NCD (Student's t-test, two-tailed).

Biology Open • Supplementary information

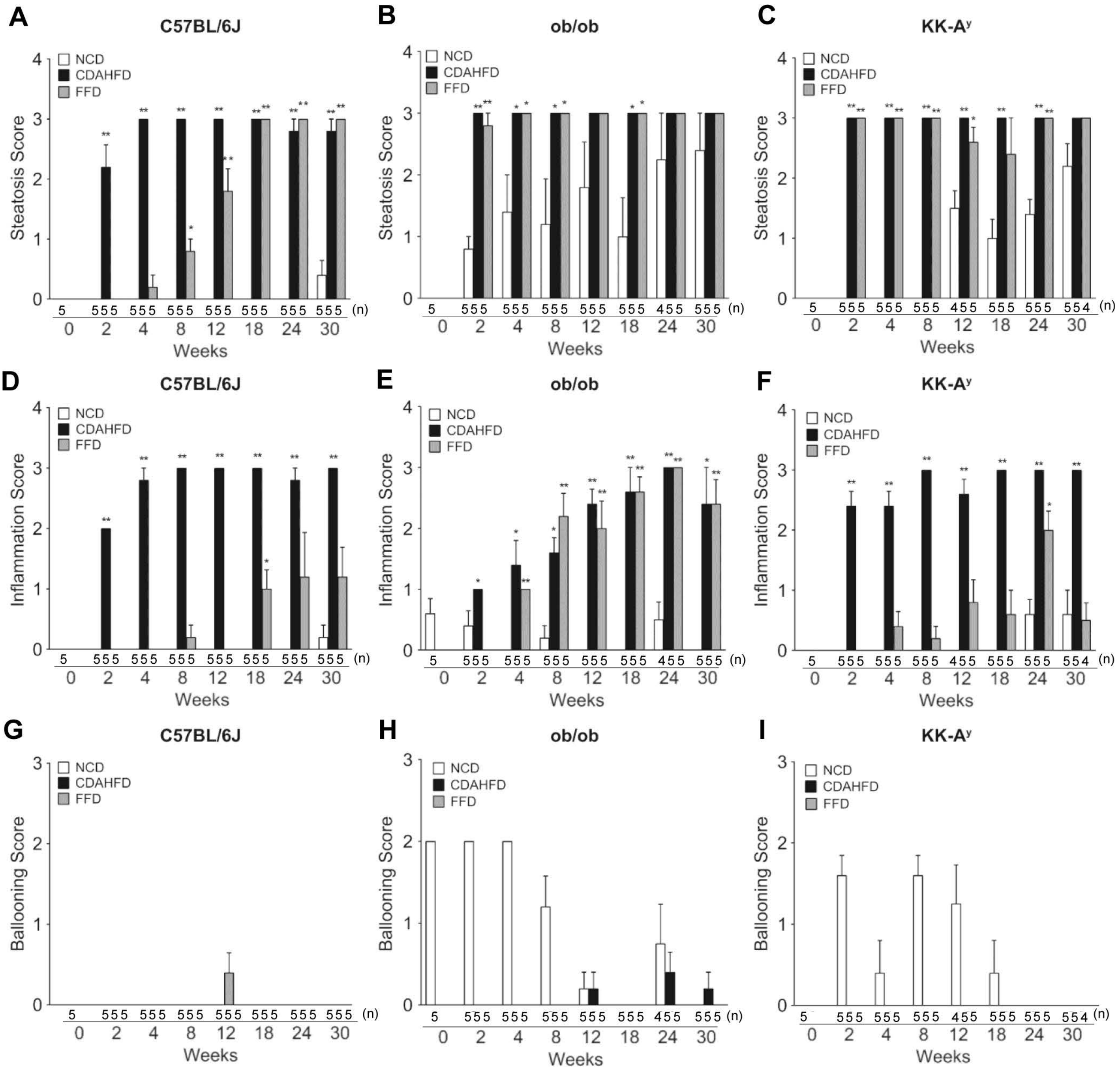

Fig. S3. Components of NAFLD activity score in C57BL/6J, ob/ob, and KK-A^y mice under NCD, FFD, or CDAHFD regimes for 2, 4, 8, 12, 18, 24, and 30 weeks. (A), (B), and (C) Steatosis score (D), (E), and (F) Inflammation score (G), (H), and (I) Ballooning score. Values are mean ± SEM, n = 4–5 (the exact number of animals are shown in the figure). $^*p < 0.05$, $^{**}p < 0.01$ vs. NCD (Wilcoxon's test).

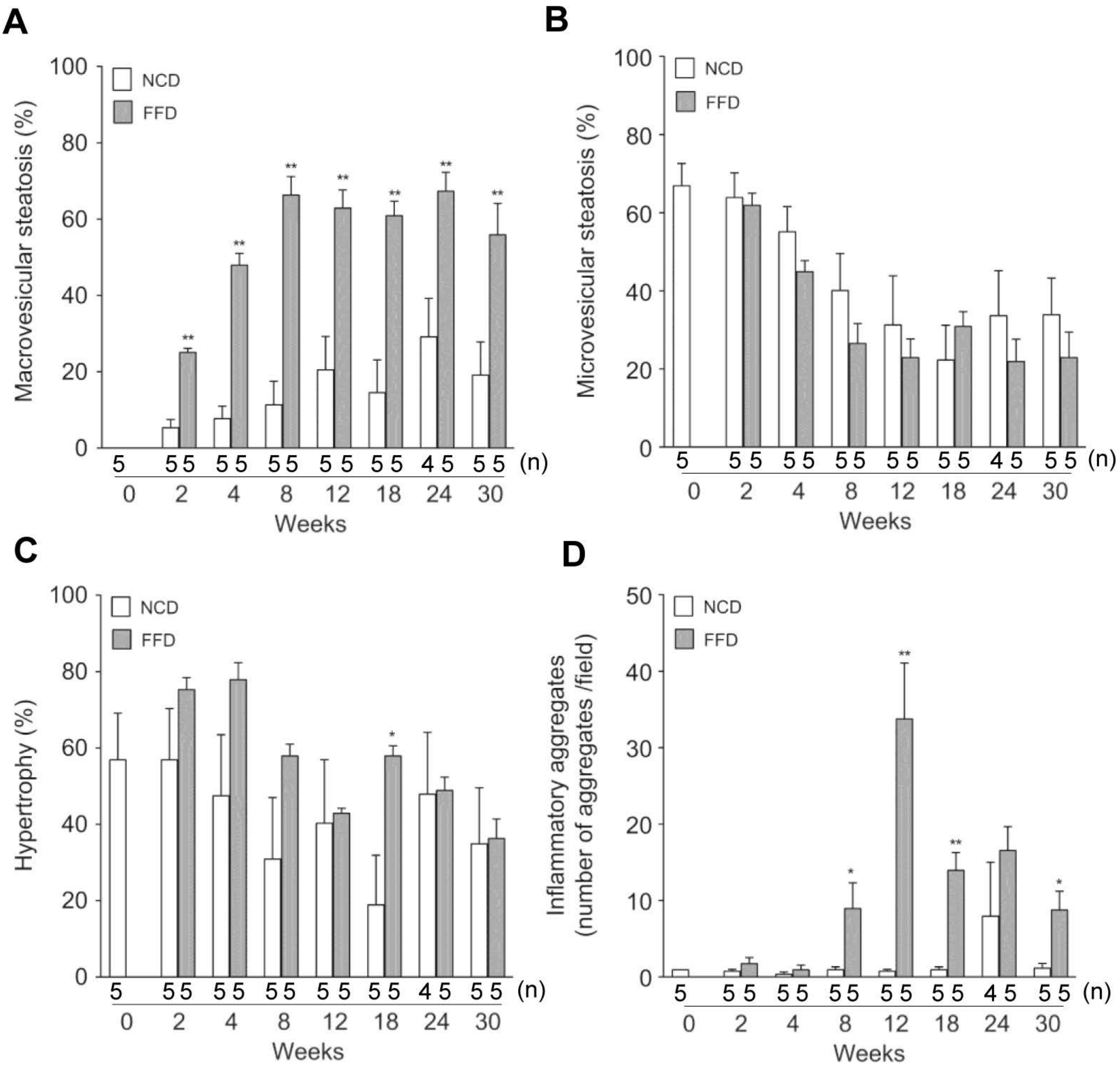

Fig. S4. Histological changes involved in human NASH histology in ob/ob mice under NCD or FFD regimes for 2, 4, 8, 12, 18, 24, and 30 weeks. (A) Macrovesicular steatosis (% surface area) (B) Microvesicular steatosis (% surface area) (C) Hepatocellular hypertrophy (% surface area) (D) Inflammatory aggregates (number of aggregates/field). Values are mean ± SEM, n = 4–5 (the exact number of animals are shown in figure). $^{*}p < 0.05$, $^{**}p < 0.01$ vs. NCD (Student's t-test, two-tailed)

Biology Open • Supplementary information
